# Potential Effects of AIT on Nonspecific Allergic Immune Responses or Symptoms

**DOI:** 10.3390/jcm12113776

**Published:** 2023-05-31

**Authors:** Kazuyuki Nakagome, Keishi Fujio, Makoto Nagata

**Affiliations:** 1 Department of Respiratory Medicine, Saitama Medical University, Saitama 350-0495, Japan; 2 Allergy Center, Saitama Medical University, Saitama 350-0495, Japan; 3 Department of Allergy and Rheumatology, Graduate School of Medicine, The University of Tokyo, Tokyo 113-8655, Japan

**Keywords:** allergen immunotherapy, IL-10-producing innate lymphoid cells, subcutaneous immunotherapy, sublingual immunotherapy, regulatory B cells, regulatory T cells

## Abstract

Allergen immunotherapy (AIT) is a treatment in which clinically corresponding allergens are administered to patients with allergic diseases, either by subcutaneous immunotherapy (SCIT) or sublingual immunotherapy (SLIT), or by oral immunotherapy (OIT) in the case of food allergy. Since etiological allergens are administered to patients, AIT is presumed to modify mainly allergen-specific immune responses. In bronchial asthma, AIT with house dust mites (HDM) alleviates clinical symptoms, suppresses airway hyperresponsiveness, and reduces medication doses of HDM-sensitive asthmatics. Moreover, AIT can suppress the symptoms of other allergic diseases associated with asthma including allergic rhinitis. However, AIT sometimes reduces allergic symptoms not induced by the responsible allergens, such as non-targeted allergens, in clinical settings. Furthermore, AIT can suppress the spread of sensitization to new allergens that are not targeted allergens by AIT, suggesting the suppression of allergic immune responses in an allergen-nonspecific manner. In this review, the nonspecific suppression of allergic immune responses by AIT is discussed. AIT has been reported to increase regulatory T cells that produce IL-10, transforming growth factor-β, and IL-35, IL-10-producing regulatory B cells, and IL-10-producing innate lymphoid cells. These cells can suppress type-2 mediated immune responses mainly through the production of anti-inflammatory cytokines or a cell–cell contact mechanism, which may be involved in the nonspecific suppression of allergic immune responses by AIT.

## 1. Introduction

Allergen immunotherapy (AIT) is a treatment in which clinically relevant allergens are administered to patients with allergic diseases [1]. AIT is effective in various types of allergic diseases such as allergic asthma, allergic rhinitis, and hymenopteran hypersensitivity [1,2]. There are subtypes of AIT, including subcutaneous immunotherapy (SCIT) and sublingual immunotherapy (SLIT), and oral immunotherapy (OIT) in the case of food allergy. SCIT or SLIT using house dust mites (HDM) improves the symptoms of asthma or allergic rhinitis and reduces medication doses in HDM-sensitive patients [3,4,5,6,7]. SCIT or SLIT using Japanese cedar pollen (JCP) suppresses the symptoms of seasonal allergic rhinitis (SAR) in JCP-sensitive patients and also inhibits JCP-related asthma exacerbation [2,8]. AIT is presumed to modify allergen-specific immune responses because etiological allergens are administered to patients.

AIT differs from pharmacological therapy in that it can modify the natural course of asthma and allergies [1]. AIT can suppress the spread of sensitization to new allergens that were not allergens originally targeted by AIT [9,10]. Furthermore, it is seen clinically that AIT can reduce allergic symptoms induced by non-targeted allergens of AIT as described below (Nakagome K, et al. unpublished observation). Therefore, AIT may suppress allergic immune responses in an allergen-nonspecific manner in some situations. In this review, the nonspecific suppression of allergic immune responses by AIT is discussed.

## 2. Mechanisms of Efficacy of AIT

AIT increases the allergen-specific generation of immunoglobulins (Igs) such as IgG, including IgG4 and IgA [11,12,13,14,15]. IgG or IgG4 suppresses the composition of allergen-IgE by competing with IgE [16]. Thus, AIT can inhibit basophil and mast cell activation by suppressing the cross linking of high-affinity IgE receptors (FcεRI). AIT can also inhibit IgE-facilitated allergen presentation from B cells to T cells by suppressing allergen-IgE binding to low-affinity IgE receptors (FcγRIIb). These mechanisms can contribute to the allergen-specific suppression induced by AIT.

AIT decreases the local Th2 cell counts or production of Th2 cytokines, such as IL-4 and IL-5 [17]. AIT inhibits the allergen-induced production of IL-5 and IL-13 [5] or that of thymic and activation-regulated chemokines from PBMCs [1]. Therefore, AIT can reduce the infiltration of Th2 cells into the airways. In addition, AIT induces regulatory T cells (Tregs) [13,15,18,19,20,21,22,23,24,25,26,27,28] and regulatory B cells (Bregs) [28,29,30,31,32], as discussed below. In addition, AIT increases the cutaneous expression of IL-12 mRNA by allergen challenge. Collectively, AIT suppresses T-cell-induced allergic immune responses by inhibiting Th2 responses and enhancing Treg or Th1 responses.

The elucidation of predictive biomarkers of AIT efficacy is of great importance. The principal candidate predictive biomarkers include the ratio of serum-specific IgE to total IgE before treatment, change in the serum allergen-specific IgG4 concentration, change in IgE-facilitated antigen binding, and change in basophil activation markers [33,34].

## 3. AIT Induces Regulatory Cells Such as Tregs and Bregs That Can Work for Nonspecific Suppression of Allergic Immune Responses

AIT induces Tregs, a heterogeneous subset of CD4^+^ T cells with immunosuppressive function that play roles in the maintenance of immune homeostasis and suppression of inflammation [27,28]. Tregs include natural Tregs that express the transcription factor forkhead box P3 (Foxp3) and inducible Tregs that produce IL-10, transforming growth factor (TGF)-β, and IL-35 [13,15,18,19,20,21,22,23,24,25,26,27,28]. Tregs suppress the functions of effector T cells and antigen-presenting cells (APCs) by various mechanisms [27,28]. For example, Tregs produce inhibitory cytokines such as such as IL-10, TGF-β, and IL-35, which are involved in the suppression of T cell differentiation and activation, suppression of cytokine production by T cells, and induction of regulatory cells such as IL-10- or IL-35-producing Tregs. Latent TGF-β with glycoprotein A repetitions predominant on the surface of Tregs inhibits T cell activation either by TGF-β1 activation or by cell-cell contact mechanism [35]. Furthermore, the ectonucleotidase CD39 and CD73 on Tregs can induce the metabolism of ATP to AMP and produce adenosine, which has an immune regulatory property. Tregs can suppress T cell proliferation by IL-2 deprivation, as IL-2 plays an important role in the survival of Tregs. Tregs also secrete granzymes and produce galectin-1, which can induce the cytolysis of effector T cells. Moreover, Tregs suppress T cell activation by suppressing the antigen-presenting capacities of APCs, including dendritic cells (DCs). Surface molecules including programmed death (PD) 1, cytotoxic T-lymphocyte antigen (CTLA) 4, lymphocyte-activation gene (LAG) 3, and inducible costimulatory molecule (ICOS) are involved in this process. CTLA-4 on Tregs inhibits the expression of CD80 and CD86 of DCs, and thus suppresses APC functions and T cell differentiation. LAG3^+^ Tregs are reported to be one of IL-10-producing Tregs [36].

AIT increases local (or peripheral) inducible Tregs, including Foxp3^+^ T cells [18,19,20], IL-10^+^ T cells [13,20,21,22,23], TGF-β^+^ T cells [15,24], and IL-35^+^ T cells [25,26]. For example, Radulovic et al. reported that grass pollen AIT increases Foxp3-expressing CD4^+^ CD25^+^ cells in the nasal mucosa [18]. In the AIT group, 20% of CD3^+^ CD25^+^ cells expressed Foxp3, and 18% of Foxp3^+^ CD3^+^ cells expressed IL-10 [18]. Furthermore, several reports suggested that increases in Foxp3^+^ T cells or IL-10^+^ T cells are related to improvements in clinical scores [20,22,23]. Terada et al. reported that JCP-SLIT increases Foxp3^+^ T cells or IL-10^+^ T cells in peripheral blood, and the number of Foxp3^+^ T cells, but not of IL-10^+^ T cells, which correlates with improvements in nasal symptoms [20]. Although natural Tregs have been shown to exhibit suppressive effects in an antigen-nonspecific manner, inducible Tregs are activated by antigen stimulation and thereby thought to suppress in an antigen-specific manner [27,37]. However, if inducible Tregs are activated by antigen, i.e., targeted allergens by AIT, they can suppress an antigen-nonspecific immune response, although it is restricted to the tissue where they are activated [27]. If not only targeted allergen by AIT but also different allergen are presented on the same DC, activated Treg specific to targeted allergen may have suppressive effect on different allergen. Rigas et al. reported that induced Tregs, but not natural Tregs, effectively suppress the production of type-2 innate lymphoid cells (ILC2)-driven IL-5 and IL-13 both in vitro and in vivo, and ICOS: ICOS–L cell contact is essential for Treg-mediated ILC2 suppression [38]. Therefore, Tregs, if activated, can induce allergen-nonspecific immune suppression through various mechanisms such as the production of anti-inflammatory cytokines or cell–cell contact.

Furthermore, several studies have emphasized the role of Bregs in the immune suppression by AIT [28,29,30,31,32]. Bregs play an important role in producing inhibitory cytokines such as IL-10, IL-35, and TGF-β, and expressing receptors that have immune suppressive properties such as PDL-1, ICOS-L, and aryl-hydrocarbon receptor [29]. Bregs are induced by several factors such as cytokines including IL-6 and interferon-α, TLR4 or TLR9 ligands, with CD40 ligation [30]. AIT increases IL-10-producing Bregs which produce IgG4 [32,39,40]. Layhadi et al. recently reported that AIT by depigmented-polymerized Phleum pratense (DPG-POL-Phl p) induces IL-10^+^ CD19^+^ CD5^hi^ and IL-10^+^ CD19^+^ CD5^hi^ CD38^int^ CD24^int^ Breg subsets, with upregulation of CD52 on Tregs [41]. Therefore, Bregs are also involved in the development of immune tolerance by AIT and can contribute to the nonspecific suppression of immune responses.

The role of T follicular helper (Tfh) cells in the induction of allergic diseases has recently been highlighted [42]. Tfh cells are responsible for B cell survival and plasma cell differentiation through the production of IL-4 and IL-21, and thus they enhance IgE-mediated allergic response [42]. Layhadi et al. reported that AIT by DPG-POL-Phl p reduces the ability to produce IL-4^+^ Tfh and IL-21^+^ Tfh cells [41]. There are also regulatory cells in Tfh cells, such as T follicular regulatory (Tfr) cells, which express FoxP3, and IL-10-producing Tfh cells. Sharif et al. reported that SCIT or SLIT induced Tfr cells and IL-10-producing circulating Tfh cells, although it suppressed circulating Tfh cells [43]. Therefore, Tfr cells and IL-10-producing Tfh cells may also play roles in the immune suppression by AIT.

Moreover, the role of innate immune responses in AIT has prevailed [44,45,46,47,48,49,50,51]. ILCs are representative cells in the system of innate immune responses, which are divided into two subsets; cytotoxic ILCs and helper ILCs. Cytotoxic ILCs include NK cells, which exhibit CD8^+^ cytotoxic T cell function. Helper ILCs were classified into three distinct phenotypes: ILC1, ILC2, and ILC3, which resemble the functions of Th1, Th2, and Th17 cells, respectively. AIT suppressed the ratio of ILC2- and IL-13-expressing ILC2 in peripheral blood [44,45,46,47]. For example, Lao-Araya et al. reported that grass pollen SCIT suppressed seasonal increases in ILC2 in the peripheral circulation. The proportion of ILC2s (Lin^−^ CD127^+^ CRTH2^+^ cells) increased by 58% in the pollen scattering season in patients with complicated SAR without SCIT, but not in those with SAR treated with SCIT [44]. Furthermore, CD117^+^ ILC2 and IL-13-expressing ILCs were also increased in the pollen scattering season in patients with complicated SAR without SCIT, whereas they did not increase in patients with SAR treated with SCIT [44]. Palomares et al. reported that SLIT using Pru p 3-enriched extracts (SLIT-Pru p 3) inhibited the frequency of ILC2 in the PBMCs of patients with lipid transfer protein allergy [45]. It also suppressed the frequency of IL-4^+^ and IL-13^+^ ILC2 in the PBMCs of patients with a lipid transfer protein allergy. In contrast, AIT can increase the ratio of ILC1 and ILC3 [45]. Eljaszewicz et al. reported that birch or grass SCIT increased the ratio of ILC1 and induced CD127^+^ CD25^+^ clusters in ILC1 observed shifts in the heterogeneity of ILC1 [48]. It also induced the CD127^+^ CD25^++^ c-Kit^+^ clusters in ILC3s [48].

**Figure 1:**
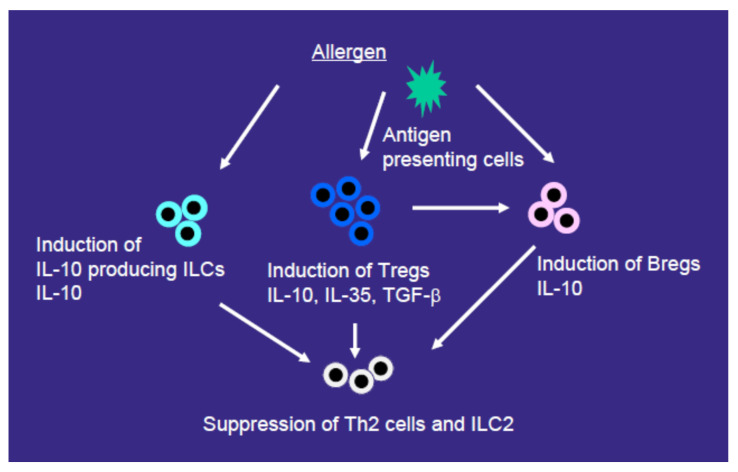
Possible mechanisms of the effects of allergen immunotherapy (AIT) for nonspecific suppression of allergic immune responses. AIT increases Tregs that produce interleukin (IL)-10, transforming growth factor (TGF)-β, and IL-35, IL-10-producing Bregs, and IL-10-producing ILCs. These cells can suppress type-2-mediated immune responses mainly through the production of anti-inflammatory cytokines or cell–cell contact mechanisms.

IL-10-producing ILCs which are induced by retinoic acid have recently been reported [49]. AIT induces IL-10-producing ILCs that can work for the allergen-nonspecific suppression of type-2-mediated immune responses [50,51]. Golebski et al. reported that killer cell lectin-like receptor G1 (KLRG1)^+^ ILC2s, but not KLRG1^–^ ILC2s, produced IL-10 after stimulation with IL-33 and retinoic acid. IL-10 production from KLRG1^+^ ILC2s after in vitro stimulation was lower in allergic patients than in healthy individuals; however, grass pollen SLIT restored IL-10 production from KLRG1^+^ ILC2s, which was inversely correlated with symptom severity [50]. Furthermore, Boonpiyathad et al. reported that IL-10^+^ CTLA4^+^ ILCs increased and the ratio of IL-4^+^ CRTH2^+^ to IL-10^+^ CTLA4^+^ ILC, decreased in the PBMCs in responders to HDM-SCIT treatment [51]. Moreover, birch or grass SCIT reduced HRA-DR^+^ intermediate monocytes and CD1c^+^ myeloid DCs [48]. AIT alters the composition and heterogeneity of innate immune cells in an allergen-nonspecific manner, suggesting trained immunity and tolerance. Therefore, anti-inflammatory cytokines such as IL-10 and IL-35, and cells that have suppressive properties such as Tregs, Bregs, and IL-10-producing ILCs, may contribute to the eventual manifestation of inhibitory effects by AIT on non-targeted allergen-induced type-2 immune responses (Figure 1).

## 4. AIT Modifies the Natural Course of Allergic Disease

AIT, unlike other pharmacological therapies, can alter the natural course of allergic disease. First, the effects of AIT persist for several years even after treatment is discontinued. For example, in the case of hay fever, 3- or 4-year AIT provides freedom from symptoms for 3 years following the cessation of treatment [52]. Moreover, 3-year AIT for rhinoconjunctivitis suppresses symptoms and allergen-challenge-induced conjunctival responses for 7 years following the cessation of treatment [53]. Furthermore, in pediatric asthmatic patients with allergic rhinitis, 5-year AIT induces maintained asthma remission for 5 years following the cessation of treatment [54].

New allergen sensitization is commonly observed in patients with allergic asthma; AIT suppresses additional allergen sensitization [9,10]. Malonia et al. reported that allergic patients without AIT were all sensitized to new allergens for 15 years (100%), whereas additional allergen sensitization was observed in 12–21% of those treated with AIT [9]. AIT also has a suppressing effect on the development of asthma in children with allergic rhinitis. In children with hay fever, 3-year AIT reduces the risk of the onset of asthma [55]. Moreover, this effect has been maintained for 7 years following the cessation of AIT. This study indicated that AIT may reduce the possibility of developing asthma when started for allergic rhinitis.

## 5. Potential Clinical Effects of AIT on Nonspecific Allergic Immune Responses or Symptoms

Allergen avoidance is another important strategy for controlling allergen-induced immune response or allergen-induced symptoms. Nishioka et al. examined the effect of home environment control on asthma exacerbation and treatment in children with asthma. They found that home environmental control targeting HDM suppresses the frequency of asthma exacerbation and dosage of treatment in HDM-sensitive childhood asthma [56]. Furthermore, surprisingly, the environmental control targeting HDM also suppresses the frequency of asthma exacerbation and dosage of treatment even in patients with non-atopic asthma who were not sensitized to HDM [56], suggesting that controlling the specific allergen-induced immune response may contribute to inhibiting the overall allergic response, including the non-targeted allergen-induced allergic response.

Furthermore, it is sometimes clinically observed that AIT attenuates allergic symptoms induced by non-targeted allergens. For example, we observed that approximately 50% of patients who have been treated with SCIT using either HDM alone or JCP alone for more than 20 years and used to have JCP-induced seasonal rhinitis as well as HDM-induced perennial rhinitis do not require drugs including nasal corticosteroid or histamine 1 receptor blocker throughout the year (Nakagome K et al. unpublished observation). Considering that the remission rate of allergic rhinitis is 13–36% in ten years [57], we think that SCIT may have some effect on symptoms induced by non-targeted allergens in some situations. In addition, AIT is known to improve the natural course of allergic diseases, such as the suppression of the spread of new allergic sensitizations, described above.

We observed that SCIT, either by HDM or JCP, suppressed non-targeted allergen (JCP or HDM)-induced production of type 2 cytokines from PBMCs from HDM- and JCP-sensitized patients (Nakagome K et al. submitted), supporting the idea of the nonspecific suppression of allergic immune responses by AIT.

As for the effect of JCP-AIT on Japanese cypress pollinosis, half of Japanese cypress pollinosis patients improve clinically with JCP-AIT. Cry j 1 and Cry j 2 are major allergens in JCP, and Cha o 1, Cha o 2, and Cha o 3 are major allergens of Japanese cypress pollen. Kikuoka et al. reported that JCP-SLIT suppresses not only Cry j 1-induced, but also Cha o 1- or Cha o 3-induced IL-5 production by PBMCs obtained from JCP and Japanese cypress pollen-sensitized patients [58]. However, these effects are thought to be due to strong amino acid sequence homology between allergens of JCP such as Cry j 1 and allergens of Japanese cypress pollen such as Cha o 1, by which the homology is different from the relationship between HDM and JCP. JCP-SLIT increases Cry j 1-induced IL-10 production from PBMCs, whereas it does not increase Cha o 1- or Cha o 3-induced IL-10 production [58]. Furthermore, JCP-SLIT increases Cry j 1-specific IgG4, but not Cha o 1- or Cha o 3-specific IgG4 in serum [58]. These differences may contribute to the findings that only half of Japanese cypress pollinosis patients improve clinically with JCP-AIT.

## 6. Conclusions

AIT may suppress not only allergen-specific but also allergen-nonspecific immune responses in some situations. AIT increases Tregs that produce IL-10, TGF-β, and IL-35, IL-10-producing Bregs, and IL-10-producing ILCs. These cells can attenuate type-2-mediated immune responses mainly through the production of anti-inflammatory cytokines or cell–cell contact mechanisms. These beneficial mechanisms may contribute to the effect of AIT on non-targeted allergen-induced type-2 immune responses or the spread of new allergen sensitizations. As the clinical evidence for allergen-nonspecific immune responses induced by AIT is currently limited, these need to be investigated in future studies of a certain scale size.

## Data Availability

Not applicable.
